# Genetic and Environmental Influences on Fetal Growth Vary during Sensitive Periods in Pregnancy

**DOI:** 10.1038/s41598-018-25706-z

**Published:** 2018-05-08

**Authors:** Tsegaselassie Workalemahu, Katherine L. Grantz, Jagteshwar Grewal, Cuilin Zhang, Germaine M. Buck Louis, Fasil Tekola-Ayele

**Affiliations:** 1 Epidemiology Branch, Division of Intramural Population Health Research, Eunice Kennedy Shriver National Institute of Child Health and Human Development, National Institutes of Health, Bethesda, MD USA; 2 Dean’s Office, College of Health and Human Services, George Mason University, Fairfax, VA USA

## Abstract

Aberrant fetal growth is associated with morbidities and mortality during childhood and adult life. Although genetic and environmental factors are known to influence *in utero* growth, their relative contributions over pregnancy is unknown. We estimated, across gestation, the genetic heritability, contribution of shared environment, and genetic correlations of fetal growth measures (abdominal circumference (AC), humerus length (HL), femur length (FL), and estimated fetal weight (EFW)) in a prospective cohort of dichorionic twin gestations recruited through the NICHD Fetal Growth Studies. Structural equation models were fit at the end of first trimester, during mid-gestation, late second trimester, and third trimester of pregnancy. The contribution of fetal genetics on fetal size increased with gestational age, peaking in late second trimester (AC = 53%, HL = 57%, FL = 72%, EFW = 71%; p < 0.05). In contrast, shared environment explained most of phenotypic variations in fetal growth in the first trimester (AC = 50%, HL = 54%, FL = 47%, EFW = 54%; p < 0.05), suggesting that the first trimester presents an intervention opportunity for a more optimal early fetal growth. Genetic correlations between growth traits (range 0.34–1.00; p < 0.05) were strongest at the end of first trimester and declined with gestation, suggesting that different fetal growth measures are more likely to be influenced by the same genes in early pregnancy.

## Introduction

Fetal growth is an important determinant of health and disease in child- and adult-hood. Measures of abnormality of fetal growth are associated with perinatal morbidity and mortality, and long-term adverse health outcomes^1–5^. Complex interactions between genetic and environmental factors including fetal and parental genetic variations, maternal nutrition, and placental function play important roles in fetal growth^6,7^. Despite the knowledge that size at birth does not reflect the pattern of fetal growth *in utero*, previous genetic and non-genetic studies have primarily used birthweight as crude measure of intrauterine growth^6–11^. Studies that demonstrate genetic and non-genetic contributions to the longitudinal pattern of growth *in utero*, identifying the timing when genetic and/or environmental factors during pregnancy are most influential, are lacking.

To date, a total of 60 loci associated with birthweight have been discovered using genome-wide association studies (GWASs)^9,10,12^. About 15% of the variance in birthweight has been explained by single nucleotide polymorphisms^10^, reinforcing earlier findings on heritability estimates of birthweight that ranged from 25–31%^13,14^. It has previously been demonstrated that the combined effect of seven candidate genetic loci on birthweight variance was similar to those of maternal smoking during pregnancy^10^, and that of 59 autosomal loci was similar to the effect of maternal body mass index^12^, suggesting that genetic loci contribute considerably high variation in birthweight. Of note, five of the seven fetal loci that were associated with birthweight, as identified by the previous GWAS study^10^, were also known to influence type-2 diabetes (*ADCY5* and *CDKAL1*), adult blood pressure (*HMGA2*, *ADRB1*) and adult height (*LCORL*)^10^. These genes encode proteins with diverse functions including transcriptional regulation, adipogenesis, and spermatogenesis. The genes are broadly expressed in several tissues indicating multiple potential downstream effects in tissues (http://www.genecards.org/).

Estimates of heritability (h^2^), which measure the proportion of total phenotypic variance attributed to additive genetics^15^, can be used to measure the extent to which fetal growth variations in a population can be explained by genetic effects^16^. Twin studies are well suited for studying genetic and environmental influences on complex traits, because estimating the correlation between monozygotic (MZ) and dizygotic (DZ) twins allows measurement of the relative contributions of fetal additive genetic, shared environmental (c^2^) and non-shared environmental (e^2^) effects on the variance and covariance of fetal growth measures^16,17^.

Heritability estimates have been used to estimate the relative contributions of genetic and non-genetic factors on parameters of growth measured at birth^18,19^. In addition, several studies have shown that additive genetic effects vary at different stages of development during infancy^20–22^, childhood^23–25^, adolescence and adulthood^23,26–28^. However, there is limited understanding of the trends in fetal genetic influences on growth trajectories *in utero*. Previous studies on heritability of fetal growth found that h^2^ of fetal growth varies over gestation, but the studies were limited to fetal anthropometry measured in late gestation and evaluated estimated fetal weight only^24,29^. Evidence suggests that early life interventions can have strong effects on the cardiovascular changes that are associated with fetal growth restriction, highlighting the importance of ascertaining sensitive “window of opportunity” for intervention^30^. A comprehensive understanding of the fetal genetic and environmental influences on variance of a wide array of fetal growth measures will be pivotal to understand the pathobiology of fetal growth, to serve as a benchmark for estimating the missing heritability of previous and future genetic studies, and to inform effective targeting of biomedical interventions. Given that fetal growth is an important determinant of health and disease in the perinatal period^31^, understanding etiology of fetal growth will have important clinical implications^30,32^.

The goal of this study was to examine the relative contributions of fetal additive genetic and environmental influences on fetal growth trajectories in a prospective cohort of dichorionic twin gestations recruited through the NICHD Fetal Growth Studies project. Specifically, we estimated h^2^, c^2^, and e^2^ on estimated fetal weight (EFW), abdominal circumference (AC), humerus length (HL), and femur length (FL) at end of first trimester, mid-gestation, late second trimester, and third trimester. We also estimated pair-wise genetic correlations between the fetal growth measures to gain insights on the extent to which the same genetic factor(s) influence different fetal growth measures during the progression of pregnancy.

## Results

### Genetic heritability of fetal growth increases throughout pregnancy

**Figure 1:**
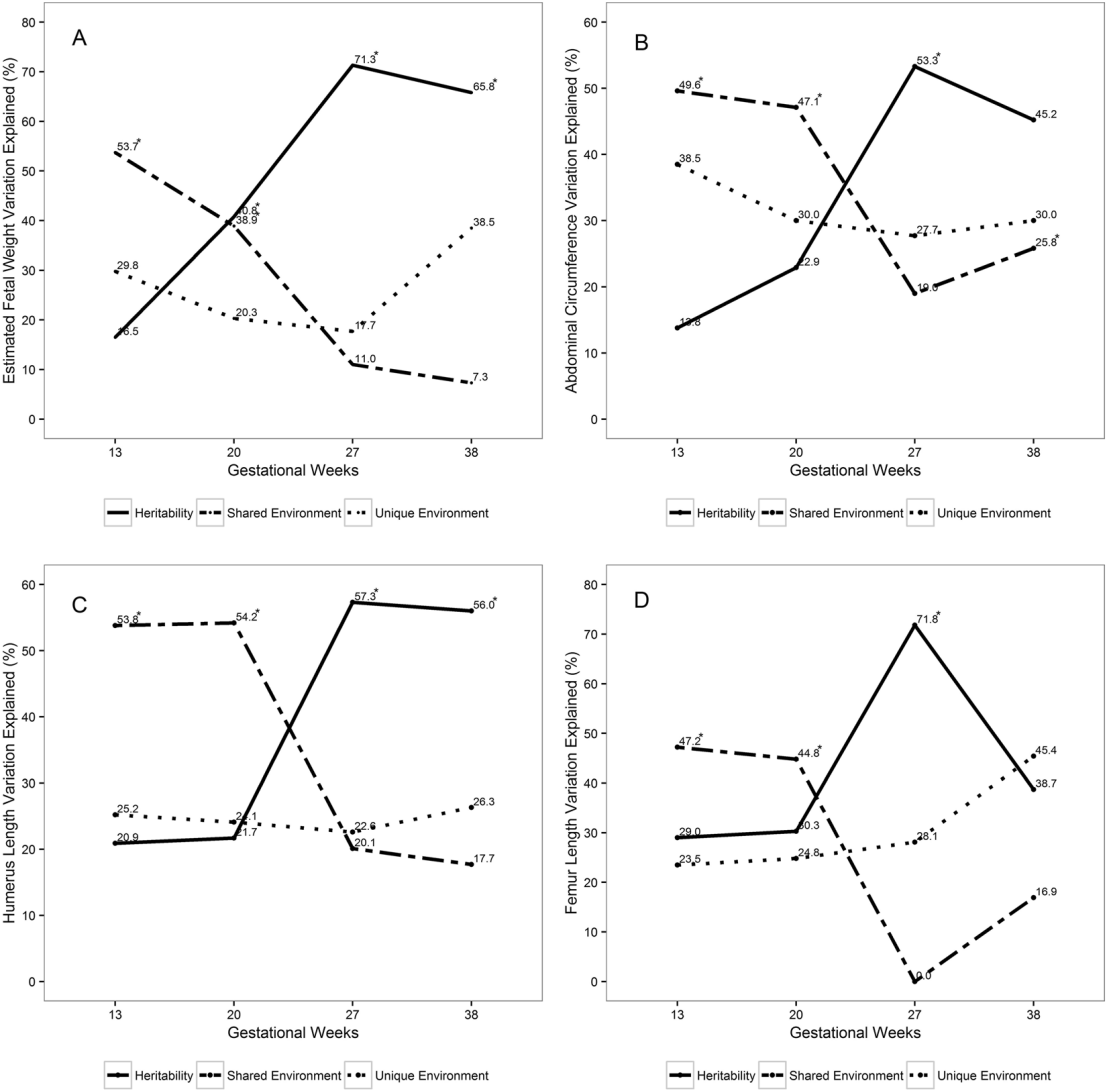
Fetal genetic heritability, shared and unique environmental variance estimates of fetal growth trajectories over gestation. (**A**) Estimated fetal weight (EFW). (**B**) Abdominal circumference (AC). (**C**) Humerus length (HL). (**D**) Femur length (FL). *Indicate statistically significant estimates (P < 0.05).

Dizygotic twins did not significantly differ from monozygotic twins with regards to their maternal and fetal characteristics and mean EFW, AC, HL and FL (Table 1). For all measures of fetal growth, h^2^ was highest in late second trimester and lowest at the end of first trimester. In contrast, c^2^ was highest at the end of first trimester and lowest in late second trimester (Fig. 1, Table S1). Specifically, h^2^ of EFW increased from end of first trimester (17%) to mid-gestation (41%), peaking in late second trimester (71%), and declining at week 38 (66%). In contrast, c^2^ declined from early through late gestation: 54% at the end of first trimester, 39% at mid-gestation, 11% at late second trimester and 7% at week 38.

**Table 1:** Study characteristics of participants.

	Monozygotic N = 15	Dizygotic N = 133	P-value^+^
**Neonatal characteristics**			
Neonatal sex (Females), n (%)	7 (46.7)	65 (48.9)	0.81
**Fetal anthropometric characteristics**			
Abdominal circumference, mm			
End of first trimester (13 weeks gestation)	76.7 ± 6.2	77.0 ± 5.0	0.77
Mid gestation (20 weeks gestation)	149.6 ± 9.4	150.5 ± 7.3	0.53
End of second trimester (27 weeks gestation)	234.1 ± 14.0	235.8 ± 9.5	0.37
Third trimester (38 weeks gestation)	328.9 ± 20.5	331.1 ± 13.7	0.43
Humerus length, mm			
End of first trimester (13 weeks gestation)	12.9 ± 1.7	13.0 ± 1.4	0.69
Mid gestation (20 weeks gestation)	30.8 ± 2.3	31.0 ± 1.9	0.56
End of second trimester (27 weeks gestation)	46.7 ± 1.8	46.9 ± 1.4	0.41
Third trimester (38 weeks gestation)	60.9 ± 2.6	61.2 ± 2.0	0.48
Femur length, mm			
End of first trimester (13 weeks gestation)	12.3 ± 1.6	12.4 ± 1.4	0.51
Mid gestation (20 weeks gestation)	31.6 ± 2.3	31.9 ± 1.9	0.33
End of second trimester (27 weeks gestation)	50.9 ± 1.7	51.3 ± 1.3	0.12
Third trimester (38 weeks gestation)	69.8 ± 2.4	70.2 ± 1.6	0.16
Estimated fetal weight, g			
End of first trimester (13 weeks gestation)	81.2 ± 9.0	82.0 ± 6.3	0.54
Mid gestation (20 weeks gestation)	329.3 ± 42.1	335.5 ± 28.3	0.28
End of second trimester (27 weeks gestation)	1124.7 ± 179.6	1157.8 ± 113.6	0.16
Third trimester (38 weeks gestation)	2933.1 ± 594.6	3046.8 ± 382.1	0.15
**Maternal characteristics**			
Maternal age, year	30.7 ± 5.4	31.8 ± 6.1	0.59
Pre-pregnancy BMI, kg/m^2^	27.7 ± 7.6	26.7 ± 6.3	0.59
Gestational age at delivery, week	34.6 ± 4.0	35.7 ± 3.7	0.28
Race/ethnicity, n (%)			0.05
White/non-Hispanic	12 (80.0)	71 (53.4)	
Other	3 (20.0)	62 (46.6)	
Parity, n (%)			0.19
0	11 (73.3)	74 (55.6)	
≥1	4 (26.7)	59 (44.4)	
Gravidity, n (%)			0.04
1	9 (60.0)	38 (28.6)	
2	2 (13.3)	48 (36.1)	
≥3	4 (26.7)	47 (35.3)	
Education, n (%)			0.24
≤ High school	1 (6.7)	25 (18.8)	
> High school	14 (93.3)	108 (81.2)	
Employment, n (%)			0.83
Employed	14 (93.3)	122 (91.7)	
Other	1 (6.7)	11 (8.3)	
Marital status, n (%)			0.20
Married	14 (93.4)	106 (79.7)	
Other	1 (6.7)	27 (20.3)	
Smoked cigarettes in the past 6 months, n (%)			0.38
Yes	1 (6.7)	20 (15.0)	
No	14 (93.3)	113 (85.0)	
Any alcohol in the past week, n (%)			0.50
Yes	0 (0.0)	4 (3.0)	
No	15 (100.0)	129 (97.0)	

^+^P-values were based on χ^2^ test for categorical variables and t-test for continuous variables.

For AC, h^2^ increased from end of first trimester (14%) to mid-gestation (23%), peaking in late second trimester (53%), but declining at week 38 (45%). In contrast, c^2^ for AC declined from end of first trimester (50%) to mid-gestation (47%), reaching 19% in late second trimester and increasing to 26% at week 38. Similar contrasting trends in h^2^ and c^2^ were observed for FL and HL. For example, h^2^ for FL slightly increased from first trimester (29%) to mid-gestation (30%), peaked in late second trimester (72%) and declined at week 38 (39%). c^2^ for FL declined from 47% at first trimester to 45% at mid-gestation, declining to 0 at late second trimester, and rising to 17% at week 38. h^2^ for HL continued to increase from 21% at the end of first trimester to 22% in mid-gestation, and 57% in late second trimester, but remained at 56% by week 38. c^2^ remained at 54% in first trimester and mid-gestation, and continued to decline to 20% at the end of second trimester and to 18% at week 38. Overall, e^2^ remained relatively similar at end of first trimester and late second trimester, except for HL and FL in which it showed an increment during the third trimester (Table S1). The corresponding p-values for h^2^ and c^2^ estimates are shown in Table S1. Maternal age, fetal sex and race were covariates that were statistically significant and explained 6.1–11.1% of variance of the fetal growth measures from the end of first trimester to end of second trimester (Table S2).

### Genetic correlation of fetal growth measures declines over gestation

Significant genetic correlations were observed between EFW and measures of skeletal growth (Table 2). The genetic correlation between EFW and FL declined from first trimester (ρ_G_ = 0.79) reaching to its lowest at week 38 (ρ_G_ = 0.67). Similarly, genetic correlation between EFW and HL continually declined from the first trimester (ρ_G_ = 0.85) reaching to its lowest at week 38 (ρ_G_ = 0.65). Similar declining trend of genetic correlations were found between AC and FL (ρ_G_ = 0.57 at first trimester and ρ_G_ = 0.35 at week 38), and AC and HL (ρ_G_ = 0.67 at first trimester and ρ_G_ = 0.39 at week 38).

**Table 2:** Genetic correlation of phenotypes and their trajectories across gestation.

	End of first trimester (13 weeks gestation)		Mid-gestation (20 weeks gestation)		End of second trimester (27 weeks gestation)		Third trimester (38 weeks gestation)	
	ρ_G_	P-value	ρ_G_	P-value	ρ_G_	P-value	ρ_G_	P-value
Abdominal circumference and humerus length	0.67	5.5 × 10^−13^	0.57	7.6 × 10^−12^	0.46	1.7 × 10^−6^	0.39	9.3 × 10^−5^
Abdominal circumference and femur length	0.57	9.1 × 10^−11^	0.40	2.1 × 10^−9^	0.47	3.2 × 10^−5^	0.35	4.1 × 10^−3^
Humerus Length and Femur length	1.00	9.9 × 10^−22^	1.00	1.0 × 10^−20^	0.90	5.2 × 10^−10^	1.00	2.7 × 10^−11^
Estimated fetal weight and femur length	0.79	6.7 × 10^−16^	0.73	1.6 × 10^−14^	0.74	1.6 × 10^−9^	0.67	4.8 × 10^−6^
Estimated fetal weight and humerus length	0.85	2.7 × 10^−18^	0.76	5.3 × 10^−17^	0.70	8.3 × 10^−12^	0.65	3.4 × 10^−8^

Models adjusted for maternal age, pre-pregnancy BMI, smoking, alcohol use, race, parity, gravidity, employment status, educational status, and fetal sex as covariates.

## Discussion

The present study estimated the heritability of fetal growth trajectories using fetal anthropometric data measured throughout gestation. To our knowledge, this is the first study that comprehensively assessed fetal genetic and environmental influences on several longitudinal fetal growth indices and identified the timing when genetic and/or environmental factors during pregnancy are most influential. We observed substantial and increasing trends of fetal genetic influences on fetal growth across gestation, where h^2^ increased from first trimester to mid-gestation and peaked in late second trimester. In contrast, we observed substantial decline in the contribution of environmental factors on fetal growth variation as gestation progresses.

A previous study found that heritability of fetal weight decreased by 23% from week 25 to week 42^29^. We observed a similar pattern, where heritability of EFW decreased by 10% from week 27 to week 38. Similar to our observation, the heritability of fetal growth in the Gielen *et al*. study^29^ peaked towards late second trimester. Likewise, in pregnancies complicated by an abnormal glucose tolerance test, genetic factors (history of a prior large-for-gestational age newborn) appeared to predict accelerated fetal growth in the late second and early third trimester (weeks 24–28)^33^. In contrast, another study reported that heritability of FL and EFW increased from second trimester onwards^24^. The investigators in that study indicated that their study may be prone to measurement error, leading to biased heritability estimates. In our study, the correlation between the expert reviewer and site sonographer was >88% for all growth parameters across visits, with 21 out of 26 measures having a correlation of ≥95%, suggesting excellent reliability^34^.

We observed that the contribution of additive fetal genetic factors to fetal growth slightly declined during the third trimester of pregnancy, whereas the variance explained by environmental factors not shared by the twin pairs showed slight increment. The third trimester is a period when the growing fetus’s demand for oxygen and nutrients is high^29^. The placenta is an important unique environment in dichorionic twins, hence a component of non-shared environmental factor with high potential to orchestrate higher growth discordance between co-twins in late gestation. Placental weight, a crude marker of placental size, has been found to be independently associated with fetal growth in the third trimester^35^. Placenta-related factors such as differences in umbilical cord insertion sites on the placenta are also known to influence fetal growth^36^. Together, these data indicate that differences between dichorionic twins in factors related to placental transport functions such as placental volume, placental mass, and site of umbilical cord attachment are likely to have stronger influence in fetal growth during this period^36–38^, explaining our observed slight increment in the contributions of shared and unshared environmental influences and lower heritability in late gestation.

Shared environmental effects comprise maternal factors including age, nutritional status, and adiposity. Several animal and human studies demonstrated the impact of these factors at different critical pregnancy time periods. Maternal third-trimester cigarette consumption was found to be a strong and independent predictor of birth weight percentile^39^. Fetuses of mothers with a higher body mass index had smaller head circumferences at early gestation (17 weeks)^40^. Maternal undernutrition and overnutrition are shown to reduce placental-fetal blood flows and stunt fetal growth in studies of animal models^41,42^. In humans, maternal undernutrition in the early stage of gestation has been linked to a number of adverse effects on fetal growth and development^43^. The animal studies showed that the critical window for programing is different among the species^41^. In our study, only maternal age and race as shared environmental factors, and fetal sex as non-shared environmental factor together explained 6.1–11.1% of variance for each of the fetal growth measures from the end of first trimester to end of second trimester. Our observation that maternal age, race and infant sex together explained the phenotypic variances may suggest that future genome-wide association studies of fetal growth may attain better power with models that adjusted for these factors.

Our findings for genetic and environmental influences of growth for twins may not be generalizable to singletons, as studies reported patterns of fetal growth differ in twins and singletons^34,44,45^. However, previous study by our group compared dichorionic twin fetuses to singletons using the current study population and found that ultrasound measured mean EFW and AC for the twins was similar to that of singletons until approximately 32 weeks^46^, consistent with other studies that compared singletons and twins. Beginning at 32 weeks of gestation, dichorionic twins had smaller EFW and AC compared to singletons. This observation for slower growth in twins compared to singletons could be due to lesser capability of sustaining adequate growth in twin fetuses throughout pregnancy^47^. In addition, maternal constraint, which involves a set of uteroplacental mechanisms by which fetal growth is restricted from reaching its genetic potential, could explain differences in growth between twins and singletons^48^.

The strong genetic correlations we observed between different fetal growth measures particularly in early gestation indicates that skeletal growth and adipogenesis may be modulated through a small set of genetic pathways in early pregnancy. Interestingly, we observed that genetic correlation was highest during the first trimester when the heritability of the fetal growth traits was the lowest. This will be useful in future genomic studies because, if a genetic variant associated with one fetal growth trait in early pregnancy is discovered, there is a high chance that the same genetic variant also influences the other correlated traits. Consistent with our finding, a recent GWAS has demonstrated significant genetic correlations between birthweight and birth length^49^. Furthermore, height and weight during infancy were found to be strongly influenced by the same additive genetic and shared environmental factors^50^.

The main strength of our study its longitudinal design and implementation of a standardized ultra-sonology protocol with established quality control. Our study population included pregnancies with dichorionic twin gestations, which allowed us to assess the influence of private environment on fetal growth (e.g. placental effects). Chorionicity is associated with adverse fetal outcomes^51–54^. A prospective study found worse outcomes for dichorionic twins^47^, while another study showed monochorionic twins had higher perinatal morbidity and mortality rates compared to discordant twins^48^. Monochorionic placentation in itself is suggested to have an inverse association with birthweight^53^. Future studies may benefit from evaluating both di- and mono-chorionic twins. While di-chorionic twins enable us to study the influence of private in-utero exposures experience by the co-twins^48^, mono-chorionic twin studies will be useful to reduce confounders in studying effects of fetal sex and genetic differences in di-zygotic twins.

Our study was underpowered to examine sex-specific genetic and environmental effects. Evaluating the sex-specific associations is important because previous studies have indicated that male and female offspring respond differently to adverse environmental exposures^55,56^. Moreover, trans-generational transmission of low birthweight linking maternal birthweight to offspring birthweight has been found to be sex-specific^57^. It should be noted that variations in the relative contribution of genetic and environmental factors on fetal growth may be due to the influence of different genetic loci at different stages of fetal growth, different levels of influence from the same locus at different gestational ages, and a combination of the two effects as well as gene-environment interactions. Lastly, we have not assessed for maternal genetic effects, and gene-gene and gene-environment interaction effects which may further elucidate mechanisms of fetal growth. Future genetic studies are needed to identify the genetic loci and pathways underlying the longitudinal heritability changes found in the present study.

In summary, additive fetal genetics explained greater proportions of phenotypic variation in fetal growth at the end of gestation. In contrast, shared environment explained most of phenotypic variation in fetal growth in the first trimester, suggesting that early pregnancy presents an intervention opportunity for a more optimal early fetal growth. Our observation for contrasting trends in genetic heritability and shared environment variance for fetal growth across gestation suggests that environmental factors have stronger influence on growth at early gestation, but are overtaken by genetic influences in late gestation. Our observation for strong genetic correlations between different fetal growth measures suggest that the same genes may influence skeletal growth, and fat mass in early gestation.

## Methods

### Study population, setting and design

The study cohort was designed from the *Eunice Kennedy Shriver* National Institute of Child Health and Human Development (NICHD) Fetal Growth Studies - twins. Briefly, a cohort of 171 (15 MZ, 133 DZ, 8 missing with same sex, and 15 missing neonatal sex and zygosity) women with dichorionic twin pregnancies was recruited from 8 clinical sites in U.S. between 2012 and 2013^34,58^. Twin pregnancies with confirmed zygosity determined using standard single tandem repeat identifier kits (Applied Biosystems AmpFLSTR Identifier PCR Amplification Kit; ThermoFisher Scientific, Waltham, MA) (15 MZ and 133 DZ) were included in this study. A standardized ultrasound protocol was implemented, and sonographers underwent extensive training and credentialing. Women underwent up to 7 ultrasound examinations at which the fetal anthropometric biometrics HC, AC, HL and FL were measured^59^. The initial ultrasound imaging was scheduled between 11 weeks 0 days and 13 weeks 6 days of gestation. Women were them randomly assigned to receive sonograms according to schedule A (16, 20, 24, 28, 32, and 35 weeks) or schedule B (18, 22, 26, 30, 34, and 36 weeks)^34^.EFW was calculated using the Hadlock formula, which incorporated HC, AC and FL^60^. Zygosity of same sex twin pairs was determined from collections of placental samples or buccal swabs using standard single tandem repeat identifier kits (Applied Biosystems AmpFLSTR Identifiler PCR Amplification Kit; ThermoFisher Scientific, Waltham, MA).

Information on sociodemographic characteristics; medical, reproductive, and pregnancy histories, and health and lifestyle behaviors was obtained through in person interviews conducted at each of the prenatal study visits as previously described^34,58^. The study was approved by the Institutional Review Boards of NICHD, participating clinical institutions, and data and imaging coordinating centers. Informed consent was obtained from all participants and the study was conducted in accordance with relevant standards and guidelines.

### Statistical analysis

Linear mixed models with a cubic spline mean structure and a random effects structure that included linear, quadratic, and cubic random effects, and an intercept term for the individual fetus within twin pair^61^, were used to model growth trajectories for twins and ascertain anthropometric measurements at 13 weeks and 6 days (end of first trimester), 20^th^ week (mid-gestation), 27 weeks and 6 days (late second trimester), and 38 weeks and 6 days of gestation (third trimester). All models included continuous variables such as maternal age, pre-pregnancy body-mass-index (BMI), and categorical variables such as smoking in the past 6 months since the time of interview, alcohol use in the past week since the time of interview, race (White/non-Hispanic vs Other), parity (nulliparous vs ≥1 child), gravidity (1, 2 or ≥3 pregnancies), employment status (employed vs other) educational status (≤high school vs >high school), and fetal sex (male vs female) as covariates. Fetal growth measure were inverse normalized to ensure that their residual kurtosis values were within normal range.

Twin studies allow us to estimate the contribution of additive fetal genetic, shared environmental and non-shared environmental factors on the variance of fetal growth measures^15,16^. MZ twins share 100% of their genes, whereas DZ twins share 50% of their genes. Both MZ and DZ twins are assumed to be sharing 100% of their shared environmental influences such as *in utero* experiences. Non-shared environmental influences, including measurement error and placenta, are assumed to be unique to the co-twins and contribute to all differences between MZ twins.

For each fetal growth measure (i.e., EFW, AC, HL, and FL), we estimated the: (1) genetic heritability, i.e. the proportion of phenotypic variance attributed to additive fetal genetic variance^15^, (2) environmental variances (shared by both twins in a pair and unique to each co-twin), and (3) genetic correlation between fetal growth measures, which measures the proportion of covariance of two traits explained by additive fetal genetics using the Sequential Oligogenic Linkage Analysis Routines (SOLAR) software version 7.2.5^62^ (http://solar-eclipse-genetics.org/). SOLAR implements a structural equation modeling approach to estimate additive genetic heritability, shared and unique environmental contributions and the best-fitting variance component models using the maximum-likelihood method^63–65^.

Our study achieved 80% statistical power to detect a 25% phenotypic variation due to additive fetal genetics, and a 50% phenotypic variation due to shared environment at α = 0.05^66^ (https://genepi.qimr.edu.au//general/TwinPowerCalculator/twinpower.cgi). Evidence for shared fetal genetic effects was estimated using ρ_G_, where pair-wise correlations were estimated using a maximum-likelihood bivariate analysis in SOLAR. Comparison of characteristics of monozygotic and dizygotic twins was done using SAS 9.4 (SAS Institute, Cary NC).

### Data availability

The datasets generated during and/or analyzed during the current study are available from the NICHD Fetal Growth Studies team or the corresponding author on request, including a short protocol with a specific research question, an analysis plan, and a completed Data Use Agreement. The data, along with a set of guidelines for researchers applying for the data, will also be posted to a data-sharing site, the NICHD/DIPHR Biospecimen Repository Access and Data Sharing [https://brads.nichd.nih.gov].

## Electronic supplementary material

Supplementary Information

## References

[1] Blair, E. in *Intrauterine growth restriction* 351–366 (Springer, 2000).

[2] Kessner, D. M. *Infant death: an analysis by maternal risk and health care*. Vol. 1 (Institute of Medicine, 1973).

[3] Marlow, N. In *Intrauterine growth restriction* 337–347 (Springer, 2000).

[4] Osmond C, Barker D, Winter P, Fall C, Simmonds S (1993). Early growth and death from cardiovascular disease in women. Bmj.

[5] Puffer, R. R. & Serrano, C. V. Patterns of birthweights (1987).

[6] Sacks DA (2004). Determinants of fetal growth. Current diabetes reports.

[7] Regnault TR, Limesand SW, Hay WW (2001). Factors influencing fetal growth. NeoReviews.

[8] Fradin D, Boileau P, Lepercq J, Bougneres P (2005). ‘Non-Mendelian’genetics of fetal growth. Journal of endocrinological investigation.

[9] Freathy RM (2010). Variants in ADCY5 and near CCNL1 are associated with fetal growth and birth weight. Nature genetics.

[10] Horikoshi M (2013). New loci associated with birth weight identify genetic links between intrauterine growth and adult height and metabolism. Nature genetics.

[11] Högberg L, Lundholm C, Cnattingius S, Öberg S, Iliadou A (2012). Birthweight discordant female twins and their offspring: is the intergenerational influence on birthweight due to genes or environment?. Human Reproduction.

[12] Horikoshi M (2016). Genome-wide associations for birth weight and correlations with adult disease. Nature.

[13] Magnus P, Gjessing H, Skrondal A, Skjaerven R (2001). Paternal contribution to birth weight. Journal of Epidemiology & Community Health.

[14] Lunde A, Melve KK, Gjessing HK, Skjærven R, Irgens LM (2007). Genetic and environmental influences on birth weight, birth length, head circumference, and gestational age by use of population-based parent-offspring data. American journal of epidemiology.

[15] Boomsma D, Busjahn A, Peltonen L (2002). Classical twin studies and beyond. Nature reviews. Genetics.

[16] Rijsdijk FV, Sham PC (2002). Analytic approaches to twin data using structural equation models. Briefings in bioinformatics.

[17] Rimfeld, K., Kovas, Y., Dale, P. S. & Plomin, R. Pleiotropy across academic subjects at the end of compulsory education. *Scientific reports* **5** (2015). 10.1038/srep11713 PMC4512149 26203819

[18] Clausson B, Lichtenstein P, Cnattingius S (2000). Genetic influence on birthweight and gestational length determined by studies in offspring of twins. BJOG: An International Journal of Obstetrics & Gynaecology.

[19] Hur Y-M (2005). A comparison of twin birthweight data from Australia, the Netherlands, the United States, Japan, and South Korea: are genetic and environmental variations in birthweight similar in Caucasians and East Asians?. Twin Research and Human Genetics.

[20] Vlietinck R (1989). Genetic and environmental variation in the birth weight of twins. Behavior genetics.

[21] Demerath EW (2007). Genetic and environmental influences on infant weight and weight change: the Fels Longitudinal Study. American Journal of Human Biology.

[22] Sovio U (2011). Association between common variation at the FTO locus and changes in body mass index from infancy to late childhood: the complex nature of genetic association through growth and development. PLoS genetics.

[23] Dubois L (2012). Genetic and environmental contributions to weight, height, and BMI from birth to 19 years of age: an international study of over 12,000 twin pairs. PLOS one.

[24] Mook-Kanamori DO (2012). Heritability estimates of body size in fetal life and early childhood. PLoS One.

[25] Silventoinen K (2007). Genetic regulation of growth in height and weight from 3 to 12 years of age: a longitudinal study of Dutch twin children. Twin Research and Human Genetics.

[26] Jelenkovic A (2016). Genetic and environmental influences on height from infancy to early adulthood: An individual-based pooled analysis of 45 twin cohorts. Scientific reports.

[27] Silventoinen K (2008). Genetic regulation of growth from birth to 18 years of age: the Swedish young male twins study. American Journal of Human Biology.

[28] Silventoinen K (2003). Heritability of adult body height: a comparative study of twin cohorts in eight countries. Twin Research and Human Genetics.

[29] Gielen M (2008). Modeling genetic and environmental factors to increase heritability and ease the identification of candidate genes for birth weight: a twin study. Behavior genetics.

[30] Crispi F, Miranda J, Gratacós E (2018). Long-term cardiovascular consequences of fetal growth restriction: biology, clinical implications, and opportunities for prevention of adult disease. . American Journal of Obstetrics & Gynecology.

[31] Barker DJ (2007). The origins of the developmental origins theory. Journal of internal medicine.

[32] Abdul‐Karim RW (1975). The clinical significance of deviations in fetal growth. International Journal of Gynecology & Obstetrics.

[33] Schaefer-Graf UM (2003). Determinants of fetal growth at different periods of pregnancies complicated by gestational diabetes mellitus or impaired glucose tolerance. Diabetes care.

[34] Grantz KL (2016). Dichorionic twin trajectories: the NICHD fetal growth studies. American journal of obstetrics and gynecology.

[35] Roland MCP (2012). Fetal growth versus birthweight: the role of placenta versus other determinants. PLoS one.

[36] Loos RJ, Derom C, Derom R, Vlietinck R (2001). Birthweight in liveborn twins: the influence of the umbilical cord insertion and fusion of placentas. BJOG: An International Journal of Obstetrics & Gynaecology.

[37] Kent EM (2011). Placental cord insertion and birthweight discordance in twin pregnancies: results of the national prospective ESPRiT Study. American journal of obstetrics and gynecology.

[38] De Paepe M, Shapiro S, Young L, Luks F (2010). Placental characteristics of selective birth weight discordance in diamniotic-monochorionic twin gestations. Placenta.

[39] Bernstein IM (2005). Maternal smoking and its association with birth weight. Obstetrics & Gynecology.

[40] Wills AK (2010). Maternal and paternal height and BMI and patterns of fetal growth: the Pune Maternal Nutrition Study. Early human development.

[41] Vuguin PM (2007). Animal models for small for gestational age and fetal programing of adult disease. Hormone Research in Paediatrics.

[42] Wu G, Bazer FW, Cudd TA, Meininger CJ, Spencer TE (2004). Maternal nutrition and fetal development. The Journal of nutrition.

[43] Coad J, Al-Rasasi B, Morgan J (2002). Nutrient insult in early pregnancy. Proceedings of the Nutrition Society.

[44] Gluckman PD, Hanson MA, Cooper C, Thornburg KL (2008). Effect of in utero and early-life conditions on adult health and disease. New England Journal of Medicine.

[45] Reece EA (1991). A prospective longitudinal study of growth in twin gestations compared with growth in singleton pregnancies. I The fetal head. Journal of ultrasound in medicine.

[46] Phillips DI (1993). Twin studies in medical research: can they tell us whether diseases are genetically determined?. The Lancet.

[47] Blickstein I, Keith LG (2004). Neonatal mortality rates among growth-discordant twins, classified according to the birth weight of the smaller twin. American Journal of Obstetrics & Gynecology.

[48] Hanson M (2014). a. & Gluckman, P. Early developmental conditioning of later health and disease: physiology or pathophysiology?. Physiological reviews.

[49] Bulik-Sullivan B (2015). An atlas of genetic correlations across human diseases and traits. Nature genetics.

[50] Van Dommelen P, De Gunst MC, Van Der Vaart AW, Boomsma DI (2004). Genetic study of the height and weight process during infancy. Twin Research and Human Genetics.

[51] Benson C, Doubilet P, Laks M (1993). Outcome of twin gestations following sonographic demonstration of two heart beats in the first trimester. Ultrasound in Obstetrics & Gynecology.

[52] Al Riyami N, Al-Rusheidi A, Al-Khabori M (2013). Perinatal outcome of monochorionic in comparison to dichorionic twin pregnancies. Oman medical journal.

[53] Papageorghiou A, Bakoulas V, Sebire N, Nicolaides K (2008). Intrauterine growth in multiple pregnancies in relation to fetal number, chorionicity and gestational age. Ultrasound in Obstetrics & Gynecology.

[54] Senoo M (2000). Growth pattern of twins of different chorionicity evaluated by sonographic biometry. Obstetrics & Gynecology.

[55] Braun JM (2011). Impact of early-life bisphenol A exposure on behavior and executive function in children. Pediatrics.

[56] Voigt M, Hermanussen M, Wittwer-Backofen U, Fusch C, Hesse V (2006). Sex-specific differences in birth weight due to maternal smoking during pregnancy. European journal of pediatrics.

[57] Ncube CN (2017). Sex-specific associations of maternal birthweight with offspring birthweight in the Omega study. Annals of epidemiology.

[58] Grewal, J. *et al*. Cohort Profile: NICHD Fetal Growth Studies–Singletons and Twins. *International Journal of Epidemiology*, dyx161 (2017). 10.1093/ije/dyx161 PMC5837516 29025016

[59] Hediger ML (2016). Ultrasound Quality Assurance for Singletons in the National Institute of Child Health and Human Development Fetal Growth Studies. Journal of Ultrasound in Medicine.

[60] Hadlock FP, Harrist R, Sharman RS, Deter RL, Park SK (1985). Estimation of fetal weight with the use of head, body, and femur measurements—a prospective study. American journal of obstetrics and gynecology.

[61] Pinheiro, J. C. & Bates, D. M. Mixed-effects models in S and S-PLUS Springer. *New York* (2000).

[62] Almasy L, Blangero J (1998). Multipoint quantitative-trait linkage analysis in general pedigrees. The American Journal of Human Genetics.

[63] Williams JT, Van Eerdewegh P, Almasy L, Blangero J (1999). Joint multipoint linkage analysis of multivariate qualitative and quantitative traits. I. Likelihood formulation and simulation results. The American Journal of Human Genetics.

[64] Kochunov P (2014). Multi-site study of additive genetic effects on fractional anisotropy of cerebral white matter: comparing meta and megaanalytical approaches for data pooling. Neuroimage.

[65] Reding-Bernal A (2017). Heritability and genetic correlation between GERD symptoms severity, metabolic syndrome, and inflammation markers in families living in Mexico City. PloS one.

[66] Visscher PM, Gordon S, Neale MC (2008). Power of the classical twin design revisited: II detection of common environmental variance. Twin Research and Human Genetics.

