# Supplementary material for: Genetic and Environmental Influences on Fetal Growth Vary during Sensitive Periods in Pregnancy
Source: Sci Rep. 2018 May 8;8:7274. doi: 10.1038/s41598-018-25706-z (PMC5940684; doi:10.1038/s41598-018-25706-z)
Supplement: Supplementary file 1 — Supplementary Information [file 41598_2018_25706_MOESM1_ESM.pdf]

Supplementary Information

Genetic and Environmental Influences on Fetal Growth Vary during Sensitive Periods in Pregnancy

Tsegaselassie Workalemahu[1], Katherine L. Grantz[1], Jagteshwar Grewal[1], Cuilin Zhang[1], Germaine M. Buck Louis[1], Fasil Tekola-Ayele[1*]
[1]Epidemiology Branch, Division of Intramural Population Health Research, *Eunice Kennedy Shriver* National Institute of Child Health and Human Development, National Institutes of Health, Bethesda, MD, USA

Correspondence:
*Fasil Tekola-Ayele, PhD
Epidemiology Branch, Division of Intramural Population Health Research,
Eunice Kennedy Shriver National Institute of Child Health and Human Development
National Institutes of Health
6710B Rockledge Drive, Room 3204
Bethesda, MD 20892-7004
E-mail: ayeleft@mail.nih.gov
Tel: 301-827-6518

**Fetal growth measures**	**Variance component (%)**	**P-value***	**Standard error**
Estimated fetal weight (13 weeks gestation)			
h^2	16.5	0.24	0.21
c^2	53.7	**5.40E-05**	0.13
e^2	29.8		0.10
Estimated fetal weight (20 weeks gestation)			
h^2	40.8	**0.03**	0.17
c^2	38.9	**0.002**	0.12
e^2	20.3		0.07
Estimated fetal weight (27 weeks gestation)			
h^2	71.3	**0.002**	0.17
c^2	11.0	0.22	0.14
e^2	17.7		0.06
Estimated fetal weight (38 weeks gestation)			
h^2	65.8	**0.02**	0.22
c^2	7.3	0.32	0.16
e^2	28.0		0.09
Abdominal circumference (13 weeks gestation)			
h^2	12.8	0.33	0.26
c^2	49.6	**0.0006**	0.16
e^2	38.5		0.12
Abdominal circumference (20 weeks gestation)			
h^2	22.9	0.19	0.22
c^2	47.1	**0.0004**	0.14
e^2	30.0		0.10
Abdominal circumference (27 weeks gestation)			
h^2	53.3	**0.03**	0.22
c^2	19.0	0.11	0.15
e^2	27.7		0.09
Abdominal circumference (38 weeks gestation)			
h^2	45.2	0.06	0.23
c^2	25.8	0.05	0.15
e^2	30.0		0.10
Femur length (13 weeks gestation)			
h^2	29.0	0.10	0.18
c^2	47.2	**1.00E-04**	0.12
e^2	23.5		0.08
Femur length (20 weeks gestation)			
h^2	30.3	0.10	0.19
c^2	44.8	**4.00E-04**	0.13
e^2	24.8		0.09
Femur length (27 weeks gestation)			
h^2	71.8	**6.07E-09**	0.09
c^2	0.0	NA	NA
e^2	28.1		0.09

Femur length (38 weeks gestation)			
h^2	38.7	0.13	0.30
c^2	16.9	0.20	0.19
e^2	45.4		0.13
Humerus length (13 weeks gestation)			
h^2	20.9	0.17	0.19
c^2	53.8	**1.00E-05**	0.12
e^2	25.2		0.09
Humerus length (20 weeks gestation)			
h^2	21.7	0.16	0.18
c^2	54.2	**9.00E-06**	0.12
e^2	24.1		0.08
Humerus length (27 weeks gestation)			
h^2	57.3	**0.01**	0.19
c^2	20.1	8.31E-02	0.14
e^2	22.6		0.08
Humerus length (38 weeks gestation)			
h^2	56.0	**0.02**	0.21
c^2	17.7	0.12	0.15
e^2	26.3		0.09

Table S1. Genetic heritability (h^2), shared (c^2) and unique (e^2) environmental variance estimates and their trajectories across gestation. Models adjusted for maternal age, pre-pregnancy BMI, smoking, alcohol use, race, parity, gravidity, employment status, educational status, and fetal sex as covariates.

*In bold are P-values <0.05.

		End of first trimester	Mid-gestation	End of second trimester	38th week
**Estimated fetal weight**	Age	p-value=0.0005	p-value=0.0005	p-value=0.002	-
	Sex	p-value=0.0009	p-value=0.0002	p-value=0.003	-
	Race	p-value=0.03	p-value=0.02	p-value=0.03	-
	Variance explained*	10.1%	11.1%	8.4%	-
**Abdominal circumference**	Age	p-value=0.03	p-value=0.02	p-value=0.02	-
	Sex	p-value=0.02	p-value=0.04	p-value=0.04	-
	Parity	p-value=0.01	p-value=0.04	p-value=0.04	-
	Variance explained*	9.6%	10.9%	7.7%	-
**Humerus length**	Age	p-value=0.009	p-value=0.01	p-value=0.002	-
	Sex	p-value=0.008	p-value=0.002	-	p-value=0.007
	Race	p-value=0.02	p-value=0.01	p-value=0.003	p-value=0.02
	Variance explained*	6.1%	7.5%	10.3%	9.8%
**Femur length**	Age	p-value=0.007	p-value=0.003	p-value=0.02	p-value=0.02
	Sex	p-value=0.02	p-value=0.01	-	-
	Race	p-value=0.04	p-value=0.01	p-value=0.0004	p-value=0.003
	Variance explained*	6.6%	8.3%	8.2%	5.0%

Table S2. Covariates that significantly explained fetal growth variations. Models adjusted for maternal age, pre-pregnancy BMI, smoking, alcohol use, race, parity, gravidity, employment status, educational status, and fetal sex as covariates.

*Variance explained by co-variates in the final model.
